# Age-Related Differences in Corticospinal Excitability during Observation and Motor Imagery of Balance Tasks

**DOI:** 10.3389/fnagi.2016.00317

**Published:** 2016-12-23

**Authors:** Audrey A. Mouthon, Jan Ruffieux, Martin Keller, Wolfgang Taube

**Affiliations:** Movement and Sport Science, Department of Medicine, University of FribourgFribourg, Switzerland

**Keywords:** aging, postural control, balance, internal representation, mental simulation, corticospinal excitability, TMS

## Abstract

Postural control declines across adult lifespan. Non-physical balance training has been suggested as an alternative to improve postural control in frail/immobilized elderly people. Previous studies showed that this kind of training can improve balance control in young and older adults. However, it is unclear whether the brain of young and older adults is activated differently during mental simulations of balance tasks. For this purpose, soleus (SOL) and tibialis motor evoked potentials (MEPs) and SOL H-reflexes were elicited while 15 elderly (mean ± SD = 71 ± 4.6 years) and 15 young participants (mean ± SD = 27 ± 4.6 years) mentally simulated static and dynamic balance tasks using motor imagery (MI), action observation (AO) or the combination of AO and MI (AO + MI). Young subjects displayed significant modulations of MEPs that depended on the kind of mental simulation and the postural task. Elderly adults also revealed differences between tasks, but not between mental simulation conditions. Furthermore, the elderly displayed larger MEP facilitation during mental simulation (AGE-GROUP; *F*_(1,28)_ = 5.9; *p* = 0.02) in the SOL muscle compared to the young and a task-dependent modulation of the tibialis background electromyography (bEMG) activity. H-reflex amplitudes and bEMG in the SOL showed neither task- nor age-dependent modulation. As neither mental simulation nor balance tasks modulated H-reflexes and bEMG in the SOL muscle, despite large variations in the MEP-amplitudes, there seems to be an age-related change in the internal cortical representation of balance tasks. Moreover, the modulation of the tibialis bEMG in the elderly suggests that aging partially affects the ability to inhibit motor output.

## Introduction

Postural control is known to decline across adult lifespan. Non-physical balance training has been suggested as an alternative training for frail people such as older adults. Balance training using motor imagery (MI) or action observation (AO) has been shown to improve balance control in young and elderly adults (Hamel and Lajoie, 2005; Tia et al., 2010; Taube et al., 2014). The combination of MI with AO (AO + MI) has also been demonstrated as an efficient way to train for balance tasks (Taube et al., 2014). Overall, the positive effect of mental simulation (e.g., MI, AO or AO + MI) on actual task execution is most probably explained by an overlap of active brain regions between motor execution and the activation of the internal representation of motor tasks while mentally simulating the task (Jeannerod, 1994; Caspers et al., 2010; Macuga and Frey, 2012; Hétu et al., 2013; Vogt et al., 2013). In young people, it has been shown that mental simulation activates similar cerebral regions to those used during real task execution (Ehrsson et al., 2003; Iseki et al., 2008; Hétu et al., 2011; Ferraye et al., 2014). With respect to postural control, we have shown in a previous fMRI study that young adults activate brain areas implicated in postural control such as SMA, cerebellum, putamen, M1 and PMC (Taube et al., 2015) during AO + MI of a dynamic balance task. Brain activation patterns depend on the kind of mental simulation (i.e., AO + MI, MI and AO) and on the postural task (i.e., static vs. dynamic). In line with this, we found that corticospinal excitability was also modulated depending on the postural task and the kind of mental simulation performed by young subjects (Mouthon et al., 2015). To date, however, it is unknown whether the internal mental representation of balance tasks undergoes age-related changes.

It is well-accepted that aging induces supraspinal changes which influence balance control (Papegaaij et al., 2014a). In line with this, decline in postural control in the elderly is shown to be associated with an increase in corticospinal excitability (Baudry and Duchateau, 2014; Baudry et al., 2015) and a reduction in intracortical inhibition (Papegaaij et al., 2014b). These results suggest that aging induces a loss of cortical inhibition as well as “over-activation” of cortical centers. In other words, aging is accompanied by a cortical disinhibition. It was assumed that these adaptations are at least partly compensatory mechanisms in order to counteract structural damage and the decline of sensory input (Papegaaij et al., 2014a). In the past, the neural processing of MI was widely investigated and it was reported that mental simulation induces modulation of corticospinal excitability. This modulation has been shown to be task-dependent and muscle specific (for review see Guillot et al., 2012; Lebon et al., 2012). However, it is not clear whether these age-related changes also affect the internal representation of balance tasks, when subjects mentally simulate postural tasks. To our knowledge, no study has addressed this issue before.

Therefore, the aim of this study is to evaluate age-related differences in corticospinal and spinal excitability, using transcranial magnetic stimulation (TMS) and peripheral nerve stimulation (PNS) during mental simulations of different standing postures. Based on the profound differences between young and elderly subjects in both postural task execution and in their corresponding brain activities (Baudry and Duchateau, 2014; Papegaaij et al., 2014a,b, 2016a,b; Baudry et al., 2015), we assumed we would observe age-related changes in the internal representation of postural tasks. More specifically, we expected to observe greater contribution of supraspinal structure in elderly people when they perform mental simulation of balance tasks.

## Materials and Methods

### Participants

Fifteen healthy older adults (10 females) aged between 65 and 80 years (mean ± SD = 71.3 ± 4.6) with no known orthopedic, neurological, or other disorders volunteered for this study. Previous to the experiment, each participant received a document presenting the motivations of the study, as well as the explanation of the TMS and PNS method, secondary effect, exclusion criteria, approved by the ethical commission. Just before the experiment, the experimenter performed a summary of the experiment, procedure, technique and answered to every question of the participant. All participants gave written consent, in accordance with the Declaration of Helsinki, before participating in this study which was approved by the local ethics committee of the canton Fribourg (014-CER-FR). Their data was compared with the results of a group of healthy young adults (aged 27 ± 4.6, five females) from a previous study (Mouthon et al., 2015).

### Experimental Procedure

**Figure 1:**
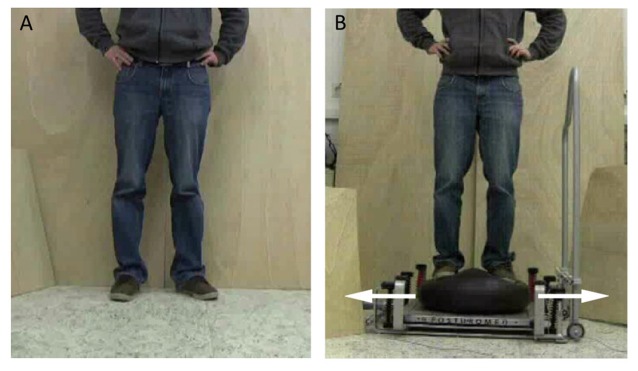
**Postural tasks presented during the experiment. (A)** For the static balance task, a person was shown standing on stable ground. **(B)** For the dynamic balance task, a person was displayed counter-balancing to compensate for a medio-lateral perturbation while standing on a free-swinging platform (from Mouthon et al., 2015).

In the course of the experiment, participants lay at rest in a supine position and looked at a monitor placed approximately one meter above their heads. Participants were instructed to execute three different types of mental simulation: (1) AO + MI, (2) MI; and (3) passive AO. For AO + MI, participants were requested to watch a video of a person carrying out a balance task. During observation of the video, participants were instructed to imagine that they were executing the task. In the MI condition, participants closed their eyes and imagined performing the balance tasks shown in the video before. During AO + MI and MI, MI was executed from a first-person perspective. For the passive AO, the instruction was to passively watch the video. Two balance tasks were displayed in the videos: (1) upright standing on stable ground (STATIC; Figure 1A); and (2) compensating for a medio-lateral perturbation while standing on a free-swinging platform (DYNAMIC; Figure 1B). Participants were introduced to the tasks and familiarized with the videos by the researcher before the experiment began.

In each of the six conditions (3 mental simulations × 2 balance tasks) the video was repeated 10 times, while 10 motor evoked potentials (MEPs) were evoked from the soleus (SOL) and tibialis anterior (TA) muscles by TMS. In order to precisely time the mental simulation, the start of each trial was signaled by three tones. The first tone was the sign to get ready. One second later, the second tone indicated that the trial would start in 2 s. The start of the trial coincided with the third tone. This was especially essential for the MI condition where participants had their eyes closed. Before and after each condition, a short rest period of 24 s was included in which participants were instructed to look at a cross on the screen. Throughout each rest period, five stimulations of TMS were applied to obtain control MEPs. Thus, there were 20 MEPs recorded for each mental simulation condition and the rest condition. The inter-stimulus interval was set to 4 s. In order to assess the spinal excitability during mental simulation, the same protocol was applied in both groups using PNS. In other words, ten SOL H-reflexes were also elicited in each condition as well as five stimulations to obtain the control H-reflex (see “Application of PNS” Section).

The six conditions were executed in a random order. This procedure was repeated twice to control for fatigue or changes in attention.

### EMG Recording

Surface electromyography (EMG) was recorded using bipolar surface electrodes (Blue sensor P, Ambu^®^, Bad Nauheim, Germany) for the SOL and TA muscles. The reference electrode was attached on the tibia plateau. The EMG signals were amplified (1000×), sampled at 4 kHz, and band-pass filtered (10–1000 Hz). Data was recorded using custom-made software (LabView^®^ based, National Instruments^®^, Austin, TX, USA).

### Application of TMS

MEPs were elicited by magnetic stimulation over the left motor cortex using a 95 mm focal “butterfly-shaped” coil (D-B80) and a MagPro X100 with MagOption magnetic stimulator (both MagVenture A/S, Farum, Denmark). The optimal site of stimulation was detected by shifting the coil until the optimal position for eliciting MEPs with minimal stimulation intensity was found. Afterwards, the coil was mechanically fixed. The location was marked on the skull in order to check whether the coil moved during the experiment. For each participant, the resting motor threshold (RMT) was determined as the lowest stimulation intensity that elicited an MEP higher than 50 μV in SOL in three out of five trials (Kujirai et al., 1993). Stimulation intensity was then set to 1.2 RMT for the experiment. It should be noted that the SOL muscle was the target muscle and stimulation intensity was adjusted to elicit MEPs in this muscle. The SOL was selected because this muscle has to act against gravity and has to be activated in order to stabilize posture after perturbation. Furthermore, H-reflexes can be elicited for the SOL muscle so that changes in Ia afferent transmission could be monitored. However, as the resting threshold is usually much higher in the SOL compared with the TA, we also recorded MEPs in the non-target muscle TA.

### Application of PNS

Electrical stimulations were delivered to the tibial nerve via an electrical stimulator (Digitizer DS7A, Hertfordshire, UK). The anode (10 cm × 5 cm dispersal pad) was placed below the patella on the anterior aspect of the knee. The cathode (2 cm in diameter) was fixed in the popliteal fossa and shifted until the optimal location to evoke a muscular response in the SOL was determined. First, a H-reflex recruitment curve was recorded by progressively increasing the stimulation intensity. When the M-wave reached a plateau, the stimulation intensity was further markedly increased in order to attain the maximal value (M-max). On the basis of the H-reflex recruitment curve, the H-reflex was adjusted to 20% of M-max and this stimulation intensity was used throughout the experiment (for trials with mental simulation and control trials). In nine out of the 15 elderly adults that were measured with TMS, distinct H-reflex responses could be elicited. These nine subjects participated in the H-reflex measurements.

### Data Analyses

In order to minimize the influence of the background EMG (bEMG) activity on trials with stimulations, trials with enhanced EMG activity preceding the MEP or the H-reflex were discarded. For this purpose, the root mean square (RMS) of the bEMG signal was calculated for a time interval of 100 ms before the stimulation. If the RMS value of a single trial reached 2.5× the standard deviation of the participant’s RMS mean, the trial was dismissed (in line with Stinear and Byblow, 2003).

Peak-to-peak amplitudes of elicited MEPs/H-reflexes were calculated. The mean amplitudes of MEP and H-reflexes were reported as the percentage of the corresponding MEPs/H-reflexes recorded during rest periods; i.e., the rest periods directly before and after each mental simulation condition.

### Statistical Analyses

Data was checked for normal distribution prior to analysis (Shapiro-Wilk test of normality). MEP/H-reflex data and bEMG were logarithmically transformed due to a skewed distribution.

For the young subjects, modulation of corticospinal excitability due to different mental simulation conditions and balance tasks was previously reported (see Mouthon et al., 2015). In congruence with this former analysis, the impact of different mental simulation conditions and balance tasks was evaluated, using a two-way repeated measures analysis of variance (ANOVA) with the factors MENTAL SIMULATION (AO + MI vs. MI vs. passive AO) and BALANCE TASK (dynamic vs. static) in the elderly subjects only.

Age-related differences in the corticospinal and spinal excitability during mental simulation were compared by means of a three way repeated measures ANOVA with the factors AGE GROUP (young vs. elderly), MENTAL SIMULATION (AO + MI vs. MI vs. passive AO) and BALANCE TASK (dynamic vs. static).

To investigate differences of bEMG between age groups, a four-way repeated measures ANOVA with the factors AGE GROUP (young vs. elderly), LEVEL OF ACTIVITY (condition vs. rest), MENTAL SIMULATION (AO + MI vs. MI vs. passive AO) and BALANCE TASK (dynamic vs. static) was performed with the RMS values.

In the case of significant main effects and/or interactions, *post hoc* Student’s *t*-tests with Bonferroni corrections were applied. A Greenhouse-Geisser correction was performed when the assumption of sphericity was violated. Data are displayed as mean ± standard error of the mean (SEM). The significance level was determined at *p* < 0.05. All statistical analyses were calculated with the software R (R Development Core Team, 2014).

## Results

### Measures of the Corticospinal Excitability

#### Elderly Adults

MEPs were excluded from statistical analysis due to increased bEMG activity prior to stimulation. On average 9% of MEPs in older adults and 5% in young adults were removed. After that, analysis of MEP facilitation of the SOL muscle in elderly adults showed a main effect of BALANCE TASK (*F*_(1,14)_ = 17.6; *p* < 0.001) with lager MEP facilitation in the dynamic task compared with the static task, but no significant main effect was found for MENTAL SIMULATION (*F*_(2,28)_ = 1.9; *p* = 0.2) and MENTAL SIMULATION × BALANCE TASK (*F*_(2,28)_ = 0.02; *p* = 0.9). Results of the non-target muscle TA were not significant: BALANCE TASK (*F*_(1,14)_ = 4.3; *p* = 0.06), MENTAL SIMULATION (*F*_(2,28)_ = 1.1; *p* = 0.3), MENTAL × BALANCE TASK (*F*_(2,56)_ = 1.1; *p* = 0.3).

#### Young Adults

Young people presented significant main effects of MENTAL SIMULATION and BALANCE TASK, as well as a significant interaction of MENTAL SIMULATION × BALANCE TASK for the SOL muscle. Results of the TA showed only a significant main effect for MENTAL SIMULATION. Details of the results for young adults are displayed in Mouthon et al. (2015).

#### Age-Related Differences

**Figure 2:**
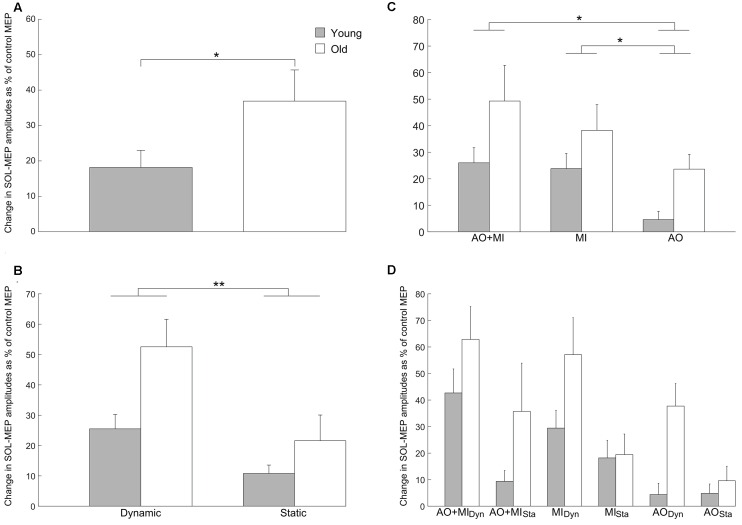
**Increase of corticospinal excitability with aging in the soleus (SOL) muscle.** Values describe percentage changes of motor evoked potential (MEP) amplitudes during mental simulation compared with control MEPs obtained at rest. **(A)** Presents the significant main effect of AGE GROUP (*F*_(1,28)_ = 5.9; *p* = 0.02) in the corticospinal excitability. **(B)** Displays the increased MEP facilitation in the elderly compared with young adults. Furthermore, it can be seen that corticospinal excitability was larger in the dynamic balance task compared with the static balance task in both groups (BALANCE TASK; *F*_(1,28)_ = 26.1; *p* < 0.001). **(C)** Presents the increase of corticospinal excitability with aging as well as the modulation of the MEP amplitudes for the three types of mental simulation in both groups (MENTAL SIMULATION; *F*_(2,56)_ = 7.4; *p* = 0.001). The interaction between mental simulation, balance task and age group is presented in **(D)**, but was not significant. Data are displayed as group mean; error bars display standard errors of the mean. Gray and white bars represent young and elderly adults, respectively. **p < 0.001, *p < 0.05.

The intensity of the TMS stimulation was not significantly different between young and older adults (55.5 ± 13.5 vs. 56.5 ± 13.7; *p* = 0.8). The comparison between young and elderly adults for the MEPs in the SOL muscle, presented in Figure 2, revealed a significant main effect of AGE GROUP (*F*_(1,28)_ = 5.9; *p* = 0.02) with larger MEP facilitation in the elderly compared with the young adults and a main effect of BALANCE TASK (*F*_(1,28)_ = 26.1; *p* < 0.001) with greater corticospinal excitability in the dynamic task compared with the static task. A main effect of MENTAL SIMULATION (*F*_(2,56)_ = 7.4; *p* = 0.001) was also significant. The interactions were not significant.

A *post hoc* test for the main effect of MENTAL SIMULATION displayed that the AO + MI condition had induced significantly stronger increases of corticospinal excitability than AO (*p* = 0.002). Similarly, the MI condition caused significantly larger MEP facilitation than the AO condition (*p* = 0.04). No significant difference in MEP facilitation was observed between the AO + MI and the MI condition (*p* = 0.9).

**Figure 3:**
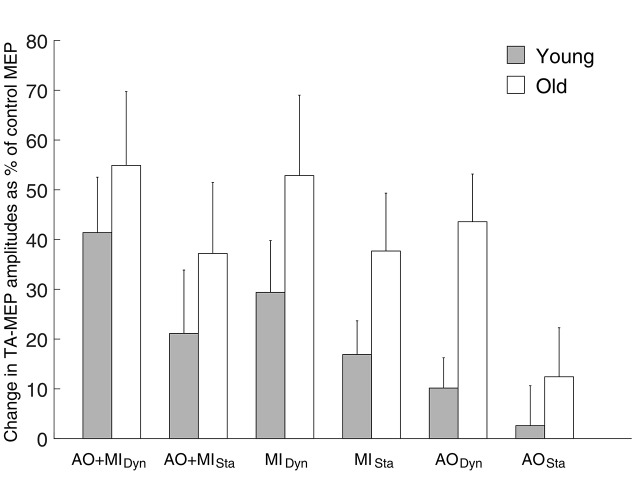
**Modulation of corticospinal excitability during mental simulation for the tibialis anterior (TA) muscle in both age groups.** Values describe percentage changes of MEP amplitudes during mental simulation compared with control MEPs obtained at rest. It can be seen that similar to the data in the SOL muscle, elderly people displayed larger MEP facilitation than young adults in all conditions. However, this effect did not reach the level of significance (*F*_(1,28)_ = 3.3; *p* = 0.07). Moreover, significant main effect of MENTAL SIMULATION (*F*_(2,56)_ = 4.1; *p* = 0.02) and BALANCE TASK (*F*_(1,28)_ = 7.8; *p* = 0.01) was revealed in both groups. Data are displayed as group mean; error bars display standard errors of the mean. Gray and white bars represent young and elderly adults, respectively.

With regard to the MEPs of the non-target TA muscle (see Figure 3), we observed a main effect of MENTAL SIMULATION (*F*_(2,56)_ = 4.1; *p* = 0.02) and BALANCE TASK (*F*_(1,28)_ = 7.8; *p* = 0.01) with greater corticospinal excitability in the dynamic task compared with the static task. The main effect of AGE GROUP was not significant (*F*_(1,28)_ = 3.3; *p* = 0.07) as was the case for all interactions (all *p* > 0.3). *Post hoc* tests revealed that only the AO + MI condition had greater MEP facilitation than the AO condition (*p* = 0.01), but no significant difference was observed between AO + MI and MI (*p* = 0.9). The MI condition showed significantly greater corticospinal excitability than AO (*p* = 0.02).

### Background EMG

Analysis of the SOL bEMG activity revealed a main effect of AGE GROUP (*F*_(1,28)_ = 92.3; *p* < 0.001). This means that muscular activity was higher in the elderly than in young subjects both at rest and during mental simulation. There was no other main effect or interaction that reached the level of significance.

**Figure 4:**
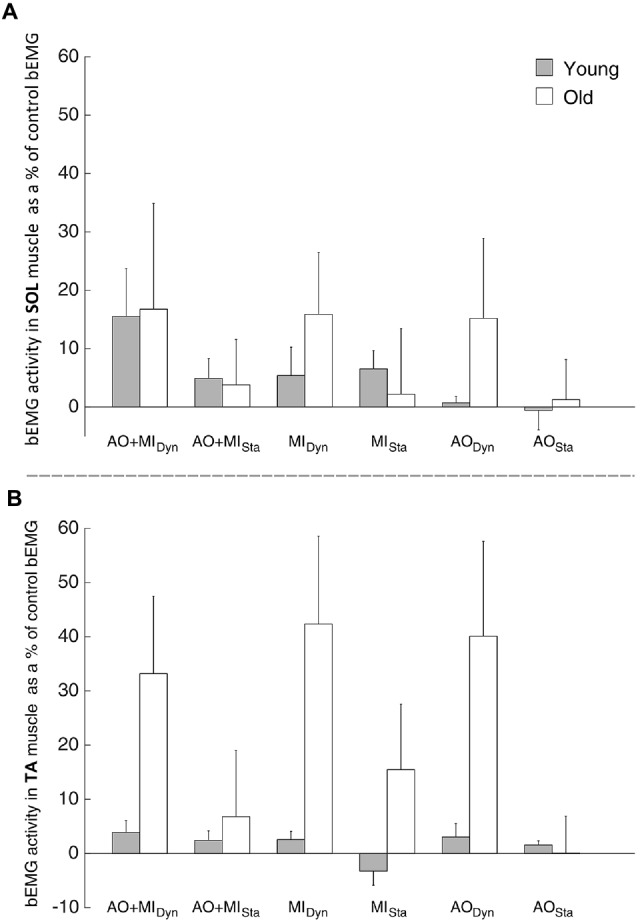
**Modulation of the background electromyography (bEMG) activity during mental simulation for (A)** the SOL and **(B)** the tibialis muscle (TA) in young and elderly subjects. Values describe percentage changes of the bEMG activity compared with the control bEMG activity during rest. Data are presented as group mean; error bars show standard error of the mean (SEM). Gray and white bars represent young and elderly adults, respectively.

Results from the TA bEMG muscle, presented in Figure 4, showed a significant main effect of AGE GROUP (*F*_(1,28)_ = 78.1; *p* < 0.001) and significant interactions of LEVEL OF ACTIVITY × BALANCE TASK (*F*_(2,56)_ = 7.5; *p* = 0.01) and AGE GROUP × LEVEL OF ACTIVITY × BALANCE TASK (*F*_(1,28)_ = 6.1; *p* = 0.02). *Post hoc* analysis showed that the bEMG was bigger when the elderly imagined the dynamic (*p* < 0.001) and static balance tasks (*p* < 0.001) than during the rest condition, compared with the young adults.

This means that muscular activity was not only higher in the elderly than in young subjects at rest and during mental simulation, but also differently modulated by the type of balance task (Figure 4).

### PNS Measures

#### Elderly Adults

For the H-reflex, elderly adults showed neither significant main effects nor significant interactions: main effect of MENTAL SIMULATION (*F*_(2,16)_ = 0.8; *p* = 0.4), BALANCE TASK (*F*_(1,8)_ = 0.05; *p* = 0.8), MENTAL × BALANCE (*F*_(2,16)_ = 1.7; *p* = 0.2).

#### Age-Related Differences

**Figure 5:**
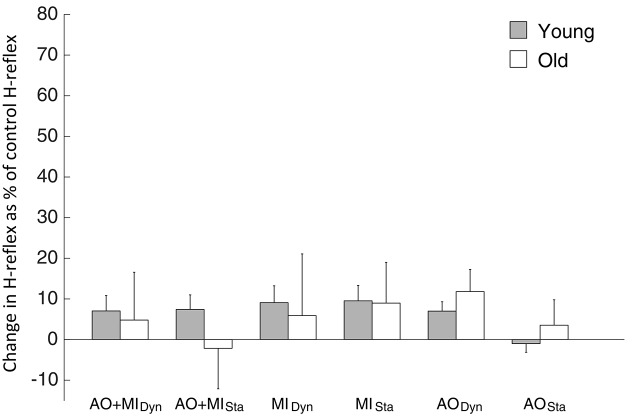
**H-reflex results observed during mental simulation for the SOL muscle in young and elderly subjects.** Values describe percentage changes of H-reflex amplitudes compared with the control H-reflex during rest. Data are presented as group mean; error bars show SEM. Gray and white bars represent young and elderly adults, respectively.

The comparison of the H-reflex between young and elderly adults did not display significant main effects: AGE GROUP (*F*_(1,18)_ = 0.3; *p* = 0.6), MENTAL SIMULATION (*F*_(2,36)_ = 0.5; *p* = 0.6), BALANCE TASK (*F*_(1,18)_ = 0.3; *p* = 0.6). Similarly, interactions were not significant (see Figure 5).

## Discussion

This is the first study to examine age-related differences in spinal and corticospinal excitability during mental simulation of postural tasks. The data shows an increase of corticospinal excitability with aging. The lack of modulation of the H-reflex and the bEMG activity of the SOL suggests that age-related changes of the internal representation of postural tasks occur at the supraspinal level. Moreover, the results for the non-target muscle TA indicate that aging may partially affect the ability to inhibit motor commands elaborated during the mental simulation of balance tasks, as aging induced modulation of bEMG activity in the TA was recorded in old but not in young participants.

### Mental Simulation in Young and Elderly People

Previous studies have demonstrated that the actual execution of balance tasks differs between young and elderly adults, not only on the behavioral but also on the neural level. Older adults demonstrate increased corticospinal excitability (Baudry and Duchateau, 2014; Baudry et al., 2015) and a reduction of intracortical inhibition (Papegaaij et al., 2014b). It was assumed that these age-related adaptations may partly serve to counteract structural damage and decline of sensory input (Papegaaij et al., 2014a). However, theoretically, decline of sensory input does not necessarily have to impair the internal representation of balance tasks. Therefore, we were interested in similarities and differences between old and young adults when mentally simulating different balance tasks.

For the young subjects, we had recently shown that MEP facilitation was more pronounced in the dynamic compared with the static task and more pronounced during the combination of AO and MI (AO + MI) than during MI and AO (Mouthon et al., 2015). The results of the elderly participants are partly in line with this previous observation: elderly people demonstrated greater corticospinal excitability in the dynamic perturbation task than in the static standing task when considering the target muscle SOL. However, when considering the type of mental simulation, elderly subjects demonstrated no significant modulation of corticospinal excitability, whereas young subjects did. Thus, although the overall pattern was similar in the elderly subjects—displaying the largest MEPs during AO + MI followed by MI and finally AO—this modulation was non-significant and less pronounced compared with the one seen in young adults. Furthermore, when directly comparing young and elderly subjects, the most obvious difference was an increased corticospinal excitability in elderly subjects compared with young subjects. Importantly, MEP-facilitation was expressed as percentage changes to the baseline values directly measured before and after the mental simulation task. Thus, there was no general over-activation in the elderly, but a task-specific one related to the mental act of imagining and/or observing balance tasks.

Importantly, this observation is very much in line with the finding of increased corticospinal excitability in elderly subjects obtained during actual task execution of postural tasks (Baudry and Duchateau, 2014; Baudry et al., 2015). Thus, age-related changes in the activation of the internal representation of balance tasks by means of mental simulation seem to reflect age-specific adaptations of the neural activity during real task execution.

It is noteworthy that the modulation of the H-reflex and the bEMG activity in the SOL muscle did not differ between young and elderly participants and were not significantly influenced by either the task or the mental simulation condition. However, it cannot be ruled out entirely that the slight and non-significant differences in bEMG between young and elderly subjects nonetheless influenced MEP-modulation. If so, a similar modulation of the H-reflex would have been expected, because it has previously been shown that SOL MEPs and H-reflexes are similarly influenced by small changes in the bEMG (Taube et al., 2008). Therefore, it seems most likely that the larger MEP facilitation in the elderly subjects during mental simulation of balance tasks is based on adaptations taking place at the cortical level. Thus, it might be assumed that in elderly participants mental simulation of postural tasks involves greater activity of neurons in the primary motor cortex. Alternatively, inhibitory processes might undergo age-related changes leading to greater corticospinal output in the elderly. About the reason for these changes can only be speculated. For the actual physical execution of balance tasks, it is assumed that the greater activation in the elderly compensates for structural degradation and implies the allocation of more neural resources to perform a balance task (Papegaaij et al., 2014a).As mental simulation seems to be able to activate the internal representations of balance tasks, the reason may just be one and the same.

### Comparison of Activity in the Soleus and Tibialis Muscle

Similar to the SOL muscle, MEP facilitation in the TA was larger during the dynamic than the static standing task. Furthermore, MEP facilitation was less pronounced in the AO condition compared with AO + MI and MI. However, although SOL and TA presented similar patterns of MEP facilitation, differences were observed. The SOL displayed significantly larger MEP facilitation in the elderly compared with young adults, whereas there was only a trend for the TA (*p* = 0.07). The reason for the lower increase in MEP facilitation with aging in this muscle may be linked to the nature of the dynamic task. As introduced in Mouthon et al. (2015), the execution of this task mainly activates the extensors to compensate for the perturbation. It might thus be presumed that the TA muscle is only slightly (co-) activated in order to stabilize the ankle joint. Age-related differences in the activity level of the TA might, therefore, not be as pronounced as in the SOL. This would be very much in line with prior studies, demonstrating that mental simulation principally affects MEP facilitation of muscles involved in the imagined or observed movement (Fadiga et al., 1999; Gangitano et al., 2001; Maeda et al., 2002).

It has to be further noted that the stimulation setup was geared to assess differences in the target muscle, SOL, so that the optimal site of stimulation, the motor threshold and the final stimulation intensity were adjusted for the SOL. Furthermore, no H-reflexes were measured in the TA, so it cannot be ruled out that changes at the spinal level contributed to the modulation of the MEP. Moreover, we found that the bEMG activity of the TA was task-specifically modulated, showing higher activity during the dynamic than during the static task. In contrast, bEMG activity in the SOL muscle was not modulated across conditions. This difference may be related to the distinct neural circuits controlling the SOL and TA muscles. Indeed, the TA muscle seems to present stronger corticospinal pathways than the SOL (Brouwer and Ashby, 1992; Bawa et al., 2002). Consequently, the elaborated motor commands for the TA may be more difficult to inhibit, especially with increasing age. It is well known that inhibitory processes undergo age-related decline (for review see Levin et al., 2014). Furthermore, mental simulation was assumed to involve elaboration of motor commands that have to be subsequently suppressed (Guillot et al., 2012). Therefore, the increased bEMG activity in the TA muscle may reflect age-related changes in inhibitory processes during mental simulation. This would indicate that aging does not only lead to a reduction of inhibition during physical balance tasks, but also decreases the ability to entirely inhibit mentally simulated motor commands. Future studies using inhibition paradigms (e.g., SICI, LICI) should explore this assumption in more detail. An alternative explanation for the increased bEMG in the TA might be that elderly subjects have a more pronounced co-activation (co-contraction) strategy to stabilize posture. It is well known that elderly subjects demonstrate greater levels of co-activation during postural task execution (Laughton et al., 2003; Benjuya et al., 2004; Donath et al., 2016). It seems likely that elderly subjects also adopt this strategy when mentally simulating postural tasks.

## Functional Considerations and Conclusion

Non-physical (mental) balance training is an interesting alternative to physical balance training for frail and/or immobilized elderly people. For that reason, it seems crucial to have a better understanding of whether mental simulation undergoes age-related changes, as this may help to adequately promote and conceptualize non-physical training for the elderly. The current study demonstrates that aging indeed induces neural changes of the internal representation of balance tasks. Moreover, the task-dependent modulation of the bEMG activity in the TA muscle indicates that elderly people partially fail to inhibit mentally simulated motor commands. Furthermore, brain activation in elderly subjects seems to be less sensitive to changes in the type of mental simulation, but still susceptible to changes in the type of balance task. Therefore, non-physical balance training with the elderly should include demanding postural tasks.

## Author Contributions

AAM contributed substantially to the acquisition, analysis and interpretation of data for this work. She also drafted the report and gave final approval of the version to be published; JR contributed to the acquisition of the data, critically revised the draft for important intellectual content and gave final approval of the version to be published; MK contributed to the conception of the work, critically revised the draft for important intellectual content and gave final approval of the version to be published; WT contributed to the conception of the study, the analysis and the interpretation of data for the study, as well as critically revising the draft for important intellectual content and giving final approval of the version to be published. He agreed to be accountable for all aspects of the work by ensuring that questions relating to the accuracy or integrity of any part of the work were appropriately investigated and resolved.

## Funding

This work was supported by the Swiss National Science Foundation (SNF research grant 320030_144016/1).

## Conflict of Interest Statement

The authors declare that the research was conducted in the absence of any commercial or financial relationships that could be construed as a potential conflict of interest.
